# Next generation diagnostic pathology: use of digital pathology and artificial intelligence tools to augment a pathological diagnosis

**DOI:** 10.1186/s13000-019-0921-2

**Published:** 2019-12-27

**Authors:** Anil V. Parwani

**Affiliations:** Division of Digital and Computational Pathology, Department of Pathology, The Ohio State University, Columbus, USA

Pathology is the study and diagnosis of disease through the examination of body tissue, which is typically fixed on glass slides and viewed under a microscope. Most medical diagnoses are made by pathologists, who, as consultants to physicians, are often referred to as “The Doctor’s Doctor”. In most labs around the world, pathology relies almost solely on glass slides to render a diagnosis. As such, initial diagnoses and subsequent second opinions are often delayed while waiting for the glass slide or specimen to be physically delivered to the appropriate pathologist and patient care may be suspended [1]. Diagnostic pathology is entering into an exciting time with the more widespread use of digital imaging in pathology, in particular, the development and deployment of whole slide imaging (WSI) technology [2]. WSI allows the scanning of entire glass slides, with an output of an image file that is a digitized reproduction of the glass slide with images that are of diagnostic quality [3, 4]. In addition, in the last 5 years we have witnessed an increasing use of machine learning (ML), deep learning (DL) and artificial intelligence (AI) tools making their way into healthcare as well as a diagnostic pathology workflow [5–7]. Thus, the timing is right for a digital disruption to occur in diagnostic pathology. The purpose of this editorial is to introduce to the readers these new and innovative tools in the diagnostic workflow and provide opportunities to bring together a series of articles on digital pathology and artificial intelligence in the next few months.

A routine (non-digital) pathology workflow usually involves the procurement of tissue, processing of tissue and creating of glass slides. The pathologist is tasked with the interpretation of glass slides locally within each hospital or a pathology group which may be serving several hospitals. This workflow is manual and requires many steps. The end result is that these glass slides are moved around and sent to pathologists to render a diagnosis [8]. This may be a time consuming and expensive process as slides are moved around, particularly if the pathologist is not directly in the local area where the slides are prepared. In some instances, specific cases are sent from the general/community pathologist to a sub-specialist/academic pathologist for an over-read consult. The role of the general pathologist is to render diagnosis at community hospitals. For cases which are challenging and difficult, these may be sent to an expert for a traditional consult, which may take days to weeks [9].

A whole slide imaging scanner may be considered a digital microscope which is outfitted with special high resolution cameras which combined with optics and software serve to produce diagnostic quality images [10]. These images are an accurate representation of the scanned glass slide and in some applications, they may be more valuable than the actual glass slides in terms of image resolution and ease of identification of specific diagnostic features [10]. Once the slides are digitized and converted to pixels, the end result is creation of a pixel pipeline which allow pathologists to remotely view the images and share them for a digital consultation [11]. Overall, it becomes much easier to share these consultation images for diagnostics as well as workflow applications. The most vital use of these digitized images is to create diagnostic algorithms or applications which can augment the diagnostic workflow. In other words, these pixels can now become part of a deep learning algorithm to look for shapes, features or patterns utilizing image analysis, deep learning and AI tools [3].

The whole slide imaging industry continues to evolve and two of the commercially available scanners are now approved by the Food and Drug Administration, which has paved the way for using WSI for primary diagnosis [12, 13]. Several studies have shown that there is minimal to no difference between a diagnosis rendered using digital images as compared to those rendered via conventional microscopy with a glass slide [2, 14–17].

Much work is still needed to digitize the pathology labs and it not as simple as changing the workflow to digital and acquire WSI scanners. Unless a fundamental change in how tissue is processed and the workflow is standardized, and the laboratory has achieved a digital workflow, computer-assisted diagnosis and automated image analysis are not possible. Several laboratories have overcome some of these above challenges and have embarked on a digital journey [18, 19].

Another advantage of a digital workflow is to reduce errors in diagnostic pathology. The incorporation of a WSI scanner in the histology lab has several advantages over conventional microscopy with an overall goal of error reduction and prevention. Once a lab is “digital, the digital images of glass slides can facilitate acquisition not only of a definitive diagnosis, but also access to other pathologists for a second opinion for quality assurance (QA). Diagnostic pathology workflow with a built in safety feature of either a digital read by second pathologist or even using a computer-aided AI algorithm to check the diagnosis that was rendered by the pathologist, especially those for suspected cancers, will enable improved patient care.

The holy grail of diagnostic pathology will be to start using these images for building helper tools for the pathologist as a decision support work flow. The images can be analyzed using DL and AI tools to look for specific features such as the number of mitotic figures, presence of infectious agents such as acid fast bacilli or even grade cancer. With the ease of availability of cloud computing, powerful processors and robust infrastructure today, it is possible to create a pixel-pipeline based workflow which allows for creating AI based prognostic or diagnostic algorithms.

Several studies in recent years have demonstrated the immense value of digital pathology and AI solutions. Beck et al. (2011) used standard anatomic pathology slides of breast cancer to train a computer algorithm to predict which patients will progress to advanced disease and which ones will not progress. The authors developed a computer algorithm to assess a set of quantitative features from breast cancer images. These measurements were then used to create a predictive model which was then used on digital images from 676 breast cancer patients. The predictive score generated by the algorithm was strongly associated with overall survival in these patients (log-rank P </= 0.001) [20]. This study successfully demonstrated an image based risk score can be generated by training the computer to detect the prognostic features only utilizing a digital image and without the need to perform expensive molecular assays that can take days to perform [20].

More recently, deep learning systems have been designed to help pathologists differentiate between benign and malignant prostate tumors, as well as the architectural patterns of prostate cancers to aid in grading of these cancers. Nagpal et al. (2019) aimed to address the issue of grading variability, improve prognostication, and optimize patient management. A two-stage deep learning system was developed to perform Gleason scoring and quantitation on prostatectomy specimens. The first stage was a deep convolutional neural network-based regional Gleason pattern classification [6]. It was trained using 912 slides with 112 million pathologist-annotated image patches. One thousand one hundred fifty-nine slide-level pathologist classifications were used for the second stage of training. A reference standard was created from the independent reviews of three pathologists as well as a genitourinary specialist pathologist on a dataset of 331 slides from 331 patients from 3 independent sources [6].

Twenty-nine additional pathologist reviewed the validation dataset as a comparison to the performance of the DLS. The mean accuracy of the 29 pathologists was 0.61 compared to an accuracy of 0.70 for the DLS (*p* = 0.002). Gleason Grade decision thresholds were also investigated with the DLS achieving area under the receiver operating characteristic curves between 0.95–0.96 at each threshold. At a Gleason Grade ≥ 4, the largest difference was seen, and the DLS showed greater sensitivity and specificity than 9 out of 10 pathologists [6].

In summary, digital pathology-based AI tools have the potential to be a disruptive technology to standard molecular and genomic based tests, which are currently believed to the only “high value” tests capable of predicting re-occurrence and therapy response outcomes.

Pathology workflow requires documentation and images are created as part of this workflow. Another utility of AI tools in diagnostic pathology is to build tools to rapidly search large image datasets to look for an image with no annotations or labels. Current digital pathology image databases often lack localized annotation of the area of interest (tumor). Many of these images are not annotated or indexed. If annotations are done, these are often limited to few features such as tumor grade, histologic subtype etc. instead of a comprehensive morphologic assessment. Digital images and AI technologies are also helping to build image search engine and helper tools for pathologists which are content based image retrieval methods [21]. Hegde et al. (2019) described AI methods developed by GOOGLE that allows for search for morphologically similar features regardless of annotation status. This algorithm was called SMILY (*Similar image search for histopatholog)* and used database of unlabeled images to find similar images [21].

As more laboratories adopt digital pathology and digital images are incorporated into the diagnostic pathology workflow there will be better case management and resulting of cases because of overall improvement in the information management surrounding a patient case. Digital pathology workflow will help improve the efficiency of reading the cases. AI tools can now be applied prior to signout of the case to help collate and integrate the information that is needed for case review but ultimately the pathologist will review the case and make a diagnosis.

Advances in digital imaging and ability to rapidly digitize glass slides has now paved the way to start using these images in deep learning/machine learning algorithms leading to potentially novel and innovative diagnostic tools. The use of DL and AI tools combined with digital pathology images can extend the value of digital pathology far beyond what is possible today and quantified above. This is the true value of digital pathology and the AI tools being developed today that will help transform diagnostic pathology.

As the editor-in-chief of Diagnostic Pathology, I am excited about the future of diagnostic medicine, particularly in the field of digital pathology coupled with artificial intelligence. We hope that our readers would enjoy the collection of articles presented in this series.

Welcome to the future of diagnostic medicine!
